# Identification of genes and alleles influencing wood development in Eucalyptus

**DOI:** 10.1186/1753-6561-5-S7-I5

**Published:** 2011-09-13

**Authors:** Simon Southerton, Shannon Dillon, Bala Thumma

**Affiliations:** 1 CSIRO Plant Industry, Australia

## 

The goal of many forest tree breeding programs is to increase the quantity and quality of wood products from plantations. Due to their outcrossing breeding systems, long generation times and relatively short history of domestication, breeding populations of most forest trees closely resemble the wild state. Consequently, vast stores of genetic variation are available for selection. Because wood traits are under polygenetic control (quantitative), genetic improvement will rely on selection of multiple alleles, each of relatively small individual effect.

Marker-assisted selection may enhance tree breeding programs by enabling informed selection of parents for crossing; fixing desirable alleles in the homozygous state; increasing selection intensity through screening large numbers of individuals; enabling early selection in seedlings and by reducing phenotyping costs. The low linkage disequilibrium found in most forest trees makes them ideally suited to candidate gene-based association mapping approaches for marker discovery. This approach seeks to find alleles which affect phenotype and that remain linked to the trait across populations and over many generations. This methodology is well suited to tree breeding programs which aim to maintain a broad genetic base i.e. programs with a large number of families.

We are using association studies to identify genes and allelic variation that influences wood fibre properties in *Eucalyptus nitens*. Candidate genes are being selected on the basis of their known involvement in cell wall synthesis pathways expected to impact wood traits. Single nucleotide polymorphisms (SNPs) are identified in candidate genes by sequencing in a number of unrelated individuals. Selected SNPs are being genotyped across large unrelated *E. nitens* populations that have been extensively phenotyped for wood properties including cellulose and lignin content, pulp yield, MFA, and density. Several SNPs significantly associated with wood properties have been identified and subsequently validated in other provenance or mapping populations growing in different environments. Selected SNPs are being investigated further to determine whether or not the SNP is the causative polymorphism and how the polymorphism influences the trait. DNA markers identified in this research may be used to complement existing index selection strategies in *E. nitens* breeding programs. Strategies for exploiting SNPs for marker-assisted selection in seedling-based breeding programs will be discussed.

